# Metformin Regulates Alveolar Macrophage Polarization to Protect Against Acute Lung Injury in Rats Caused by Paraquat Poisoning

**DOI:** 10.3389/fphar.2022.811372

**Published:** 2022-05-13

**Authors:** Ding Yuan, Yi Li, Linlin Hou, Fang Yang, Cuicui Meng, Yanwu Yu, Changhua Sun, Guoyu Duan, Zhigao Xu, Guiying Zhu, Jianjun Guo, Leilei Zhang, Gaiqin Yan, Jihong Chen, Yanan Yang, Yan Zhang, Yanxia Gao

**Affiliations:** ^1^ Department of Emergency Medicine, Henan Key Laboratory of Emergency and Trauma Research Medicine, The First Affiliated Hospital of Zhengzhou University, Zhengzhou, China; ^2^ Emergency Department, State Key Laboratory of Complex Severe and Rare Diseases, Peking Union Medical College Hospital, Chinese Academy of Medical Science and Peking Union Medical College, Beijing, China; ^3^ Academy of Medical Sciences of Zhengzhou University Translational Medicine Platform, Zhengzhou, China

**Keywords:** metformin, paraquat, acute lung injury, alveolar macrophage, polarization

## Abstract

This study explored the role of metformin (MET) in regulating the polarization of alveolar macrophages to protect against acute lung injury (ALI) in rats caused by paraquat (PQ) poisoning. The *in vivo* studies showed that the 35 mg/kg dose of MET increased the survival rate of rats, alleviated pathological damages to the lungs and their systemic inflammation, promoted the reduction of the pro-inflammatory factors interleukin-6 (IL-6) and tumor necrosis factor-α (TNF-α) levels, and increased the anti-inflammatory factor IL-10 levels in the rat serum. At the same time, the MET intervention decreased the expression of M1 macrophage marker iNOS in the lungs of the PQ-poisoned rats while increasing the M2 macrophage marker, Arg1, expression. *In vitro*, the concentration of MET > 10 mmol/L affected NR8383 viability adversely and was concentration-dependent; however, no adverse impact on NR8383 viability was observed at MET ≤ 10 mmol/L concentration, resisting the reducing effect of PQ on NR8383 vitality. The PQ-induced NR8383 model with MET intervention showed significantly reduced secretions of IL-6 and TNF-α in NR8383, and lowered expressions of M1 macrophage markers iNOS and CD86. Additionally, MET increased IL-10 secretion and the M2 macrophage markers, Arg1 and Mrcl, expressions. Therefore, we speculate that MET could regulate alveolar macrophage polarization to protect against PQ-poisoning caused ALI.

## Introduction

Paraquat (PQ), a highly effective and widely used herbicide in agricultural production, has a high mortality rate (40–60%) in the absence of any specific antidote (Gawarammana and Buckley, 2011; Huang et al., 2019; Gao et al., 2021a). Therefore, improving the survival rate of patients with PQ poisoning is a medical problem seeking an urgent solution. The PQ toxicity affects many organs within the body, the lung being the primary target, manifesting as acute lung injury (ALI) or acute respiratory distress syndrome in the early stages and pulmonary fibrosis in the later stages (Dinis-Oliveira et al., 2008; Amin et al., 2021). Acute lung injury is the leading cause of death from PQ poisoning; the primary pathophysiological mechanism being the imbalances in inflammatory responses caused by the excessive immune response, leading to the inflammatory cascade effect (Subbiah and Tiwari, 2021). At present, the drugs for treating PQ poisoning include glucocorticoids and immunosuppressants, often causing side effects such as osteoporosis and liver and kidney damage (Xu and Lu, 2019). Therefore, it is of great significance to explore new targets and new methods for PQ-poisoning immunotherapy.

Macrophages are the common immune cells in the lung, playing an indispensable role in the immune process and are also the first line of defense against foreign substances invasion (Chen et al., 2020b). Under the influence of external factors, macrophages get polarized into pro-inflammatory M1 type and anti-inflammatory M2 type, which is closely related to the occurrence and development of ALI (Laskin et al., 2019). Wang et al., (2018) reported an increase in the M1 macrophages and a decrease in the M2 macrophages induced by lipopolysaccharide (LPS) in the ALI mouse model. Sun et al., (2016) also found similar results induced by acute pancreatitis in the ALI mouse model. In the PQ-poisoning induced ALI mouse model, Wu et al., (2020) observed that the polarization of lung macrophages increased toward M1 and decreased toward M2. We (Zhang et al., 2021) also found a close relationship between ALI and the increased M1 polarization of lung macrophages in the PQ-poisoned rat model, confirming that PQ promoted the NR8383 glycolytic metabolism and enhanced their polarization to M1 type. Thus, interventions regulating glucose metabolism may become the potential therapeutic targets for attenuating ALI caused by PQ poisoning.

Metformin (MET) lowers blood sugar by regulating the body’s glucose metabolism and is considered safe (He, 2020). MET plays an important role in many diseases by regulating the immune system (Ursini et al., 2018), Yan et al., (2018) found that the ability of MET to attenuate osteolysis induced in mouse calvaria by the particles was related to the polarization of macrophages to an anti-inflammatory functional phenotype, Qing et al., (2019) reported that MET induced the M2 macrophage polarization to accelerate the wound healing. Wu et al., (2019) observed that MET had a protective effect against PQ-induced ALI. However, there are no relevant reports to confirm the relationship between the MET protective effect against PQ-induced ALI and the regulation of macrophage polarization. This study explored whether MET could regulate the polarization of alveolar macrophages, thereby attenuating the effect of PQ-induced ALI, besides exploring the new mechanism of MET attenuating PQ-induced ALI through the immune regulation perspective. The results would suggest ways to overcome the various side effects of the immunosuppressants systemic application, besides providing a new method for the precise treatment of PQ-induced ALI.

## Materials and Methods

### Reagents and Antibodies

The study used the following reagents: paraquat (Sigma-Aldrich, United States); metformin (Sigma-Aldrich, United States); hematoxylin and eosin (HE) staining reagent (Department of Pathology of Zhengzhou University, China); Rat interleukin (IL)-6 (No.70-EK306/3-96), tumor necrosis factor (TNF)-α (No.70-EK382HS-96), and IL-10 (No.70-EK310/2-96); enzyme-linked immunosorbent assay (ELISA) kits (Multi Sciences (LIANKE) Biotechnology Company, China); fetal bovine serum (Gibco, United States); F12K culture medium (Sigma-Aldrich, United States); cell counting kit (CCK-8) assays (Dojindo, Japan); bicinchoninic acid (BCA) kit (Thermo Fisher Scientific, United States); cell lysis solution (GBCBIO, Guangzhou, China); quantitative polymerase chain reaction (q-PCR) kit (TaKaRa, Dalian, China); polyvinylidene fluoride membrane (Millipore, Bedford, MA); inducible nitric oxide synthase (iNOS) (SC-7271) and arginase 1 (Arg1) (SC-271430) (Santa Cruz Biotechnology, United States); and β-actin (13E5) (Cell Signaling Technology, United States).

### Animals and Cell Lines

We purchased 100 adult male Sprague–Dawley (SD) rats, weighing 200 ± 20 g, from Beijing Vital River Laboratory Animal Technology Co., Ltd., Beijing, China. The animals were housed in a standard laboratory environment and were given a rodent animal diet and water at will. The Research and Clinical Trial Ethics Committee of the First Affiliated Hospital of Zhengzhou University approved the animal experiments (Permit number: 2019-KY-384).

Alveolar macrophages (NR8383) were purchased from the National Collection of Authenticated Cell Cultures (Shanghai, China). NR8383 were cultured in an incubator (37°C, 5% CO_2_) with the F12K medium containing 20% fetal bovine serum; the medium was changed every 2 days, and the cells were passaged 1:2 upon reaching 80% confluence.

### Survival Experiment of Paraquat-Poisoned Rats Intervened by Metformin

Sixty rats were selected and randomly divided into 4 groups: 1) normal control group (NC group, *n* = 10): 1 ml of saline was injected intraperitoneally on the left side, and 1 h later, 1 ml of saline was injected intraperitoneally on the right side; 2) PQ-poisoned group (PQ group, *n* = 10): PQ was injected intraperitoneally on the left side at a dose of 35 mg/kg, and 1 ml of normal saline was injected intraperitoneally on the right side after 1 h; 3) MET treatment group (PQ + MET group, *n* = 30): 1 h after the administration of PQ, MET was injected into the right abdominal cavity at doses of 13,25 and 35 mg/kg; 4) MET control group (MET group, *n* = 10): 1 ml of normal saline was injected intraperitoneally on the left side, and MET was used for intraperitoneal injection on the right side at a dose of 35 mg/kg after 1 h. The MET dose was administered once a day for 7 consecutive days. The death of rats in the four groups was observed and recorded.

### Establishment of Paraquat-Acute Lung Injury Model

Referring to our previous research results (Zhang et al., 2021), we selected the dose of 35 mg/kg of PQ by intraperitoneal injection to construct an ALI model of PQ-poisoned rats, and set the experimental end point at 48 h after modeling.

Forty rats were randomly divided into the following four groups: 1) NC group (*n* = 10): 1 ml of normal saline was injected intraperitoneally on the left side, and 1 h later, 1 ml of normal saline was injected into the right side intraperitoneally again; 2) PQ group (*n* = 10): PQ was diluted into 1 ml normal saline at a dose of 35 mg/kg and injected intraperitoneally on the left side; 1 h later, the right side was injected intraperitoneally with 1 ml normal saline; 3) PQ + MET group (*n* = 10): 1 h after intraperitoneal injection of PQ (35 mg/kg), MET (35 mg/kg) was used again for the right intraperitoneal injection; 4) MET group (*n* = 10): 1 ml normal saline was injected intraperitoneally on the left side, and after 1 h, MET was diluted to 1 ml normal saline at a dose of 35 mg/kg for intraperitoneal injection on the right side. The MET dosage was once a day for a total of two administrations. 48 h after the intraperitoneal injection of PQ, the rats were sacrificed, and the lungs and serum were harvested for the next steps in 10 min.

### Establishment of the Paraquat-Intervention Cell Model

NR8383 were divided into 4 groups: 1) normal control group (NC group): cells in 4 ml of medium were treated with 10 μL of PBS; 2) PQ-intervention group (PQ group): cells in 4 ml of medium were treated with 10 μL of PQ solution (PQ final concentration 10 μmol/L); 3) MET intervention group (PQ + MET group): cells in 4 ml of medium were pretreated for 1 h with 10 μL of PQ solution and then treated with 10 μL of MET solution (PQ final concentration of 10 μmol/L and MET final concentration of 5 mmol/L); 4) MET group (MET group): cells in 4 ml of medium were treated with 10 μL of MET solution (MET final concentration 5 mmol/L). The cell groups were cultured in an incubator for 48 h, harvested, and used for further experiments.

### Determination of Lung Wet/Dry Ratio

The right upper lobe lung of the rat was washed with normal saline three times, and the surface water of the lung was sucked dry with filter paper for weighing. The tissues were dried at 70°C for 72 h and weighed again to calculate the lung wet/dry weight ratio.

### Lung Histopathology

The left upper lobe lung of the rat was fixed with 4% paraformaldehyde for 72 h, then embedded in paraffin, sectioned, and dewaxed. The sections were stained with hematoxylin and eosin, and the pathological changes of lung tissue were observed under a light microscope. The severity of lung injury was graded 0–4 on four aspects: 1) alveolar congestion; 2) hemorrhage; 3) infiltration of neutrophils in the alveolar space or blood vessel walls; and 4) thickening of the alveolar walls and/or formation of a transparent film. We searched for 3 observers with a certain histopathological basis to blindly observe all lungs with HE staining and grade them according to the lung injury scoring standard (El-Sherbiny et al., 2021). The lung injury score was calculated as the sum of the score of each indicator.

### Determination of Inflammatory Factors

The samples were processed according to the instructions of the ELISA kit, blank control holes and standard holes were set during the experiment, and the absorbance value at 450 nm was measured. A standard curve was drawn with the standard absorbance value and its corresponding concentration value. The concentration value of the sample was calculated according to the standard curve, and the contents of IL-6, TNF-α, and IL-10 were detected.

### NR8383 Survival (CCK-8 Assay)

NR8383 in the logarithmic growth phase were uniformly seeded in 96-well plates (100 μL/well). The 96-well plates were kept in the incubator until the cells were attached to the wells. 1) The cells were treated with different concentrations of MET (0.5, 1, 2.5, 5, 7.5, 10, 25, 50, 75, and 100 mmol/L) for 48 h; 2) 1 h after treated with 10 μmol/L PQ, the cells were treated with different concentrations of MET (1, 2.5, 5, 7.5, 10, 25, 50, 75, and 100 mmol/L) for 48 h. A small amount (10 μL) of CCK-8 solution was added to each well. The 96-well plates were further incubated in the incubator for 3 h, and the absorbance was measured at 450 nm.

### Western Blotting Analysis

Total protein in the lung or NR8383 was extracted by radioimmunoprecipitation assay (RIPA), and quantified by the bicinchoninic acid (BCA) kit. The total protein (30 μg) was separated on a sodium dodecyl sulphate–polyacrylamide gel electrophoresis (SDS–PAGE) gel, and then transferred onto the polyvinylidene fluoride membrane. The membranes were blocked in 5% fat-free milk at room temperature for 2  h, and then incubated with primary antibodies at 4°C overnight first, followed by incubation with the corresponding secondary antibodies at room temperature for 2 h. Finally, the membranes were immersed in an enhanced chemiluminescence reagent (Thermo Fisher Scientific) in the dark and developed through an Amersham Imager 600 (GE, Fairfield, Connecticut, United States). The β-actin expression level was used as the internal control. The antibodies iNOS (1:500), Arg1 (1:1,000) and β-actin (1:1,000) have been described in the Materials section.

### Immunohistochemistry

The rat lung sections were incubated with primary antibodies specific to iNOS (1:500 dilution) and Arg1 (1:500 dilution) overnight at 4°C, and the sections were washed three times with PBS and incubated with biotinylated goat anti-mouse immunoglobulin 4°C for 60 min. The sections were then treated with streptavidin horseradish peroxidase at 37°C for 10 min, counterstained with hematoxylin, and mounted. Finally, an inverted microscope (Leica, Wetzlar, Germany) was used to examine the stained sections.

### Quantitative Real-Time Polymerase Chain Reaction

Gene expressions were analyzed by isolating total RNA using TRIzol reagent from lung tissue or NR8383, followed by reverse transcription of RNA into cDNA using the TaKaRa kit. Finally, we conducted the qRT-PCR analyses on a Deep Well Real-Time PCR Detection System (CFX96 Touch™; Bio-Rad, Hercules, CA). Each experiment was performed thrice in three replicate wells. Data were normalized to β-actin as endogenous control, and relative expressions of the target genes were expressed using the 2^−ΔΔCt^ method; Table 1 lists the used primers.

**TABLE 1:** Primer sequences used for qRT-PCR.

mRNA	Primers	Sequences (5′–3′)
iNOS	Upstream	AGACGCACAGGCAGAGGT
	Downstream	AGGCACACGCAATGATGG
Arg1	Upstream	GTG​AAG​AAC​CCA​CGG​TCT​GT
	Downstream	GCC​AGA​GAT​GCT​TCC​AAC​TG
CD86	Upstream	GCT​CTC​AGT​GAT​CGC​CAA​C
	Downstream	TCT​TTG​TAG​GTT​TCG​GGT​ATC
Mrc1	Upstream	CTT​CGG​GCC​TTT​GGA​ATA​AT
	Downstream	TAG​AAG​AGC​CCT​TGG​GTT​GA
β-actin	Upstream	ACT​ATC​GGC​AAT​GAG​CGG​TTC​C
	Downstream	CTG​TGT​TGG​CAT​AGA​GGT​CTT​TAC​G

### Statistical Analysis

The statistical data were analyzed using SPSS26.0 software (IBM). We used the Kolmogorov–Smirnov method to test normality, and the normal distribution data were expressed as mean ± standard deviation. An independent Student’s *t*-test was used to compare two groups, while one-way analysis of variance (ANOVA) was used to compare more than two groups. *p* < 0.05 was considered statistically significant.

## Results

### Metformin Increased the Survival Rate of Rats After Paraquat Poisoning

**FIGURE 1:**
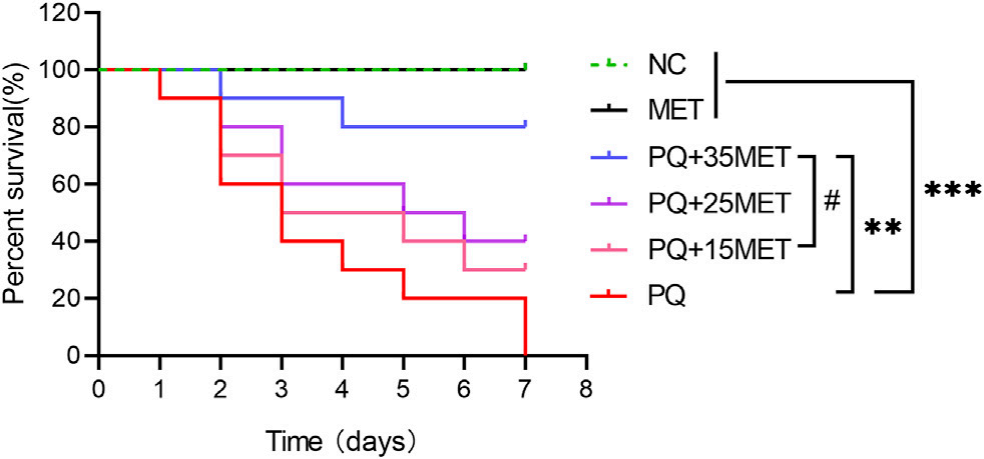
Effect of MET on the survival rate of PQ-poisoned rats. MET at doses of 15, 25 and 35 mg/kg was used to treat PQ-poisoned rats, and the survival of rats in each group was recorded (*n* = 10). Statistical analysis between groups were conducted, ^#^ *p* < 0.05, ^**^ *p* < 0.01, ^***^ *p* < 0.001.

As shown in Figure 1, on the seventh day after the start of the experiment, all the rats in the PQ group died, compared with the NC and MET groups, there was a significant difference (*p* < 0.001). MET doses of 15, 25 and 35 mg/kg had a dose-dependent increase in the survival rate of rats after PQ poisoning, the survival rate of the rats in the PQ+35MET group was higher than that in the PQ+15MET group (*p* < 0.05). However, compared with the survival rate of the rats in the PQ group, only the PQ+35MET group had significant statistical significance (*p* < 0.01).

### Metformin Alleviated Acute Lung Injury and Systemic Inflammation in Rats After Paraquat Poisoning

**FIGURE 2:**
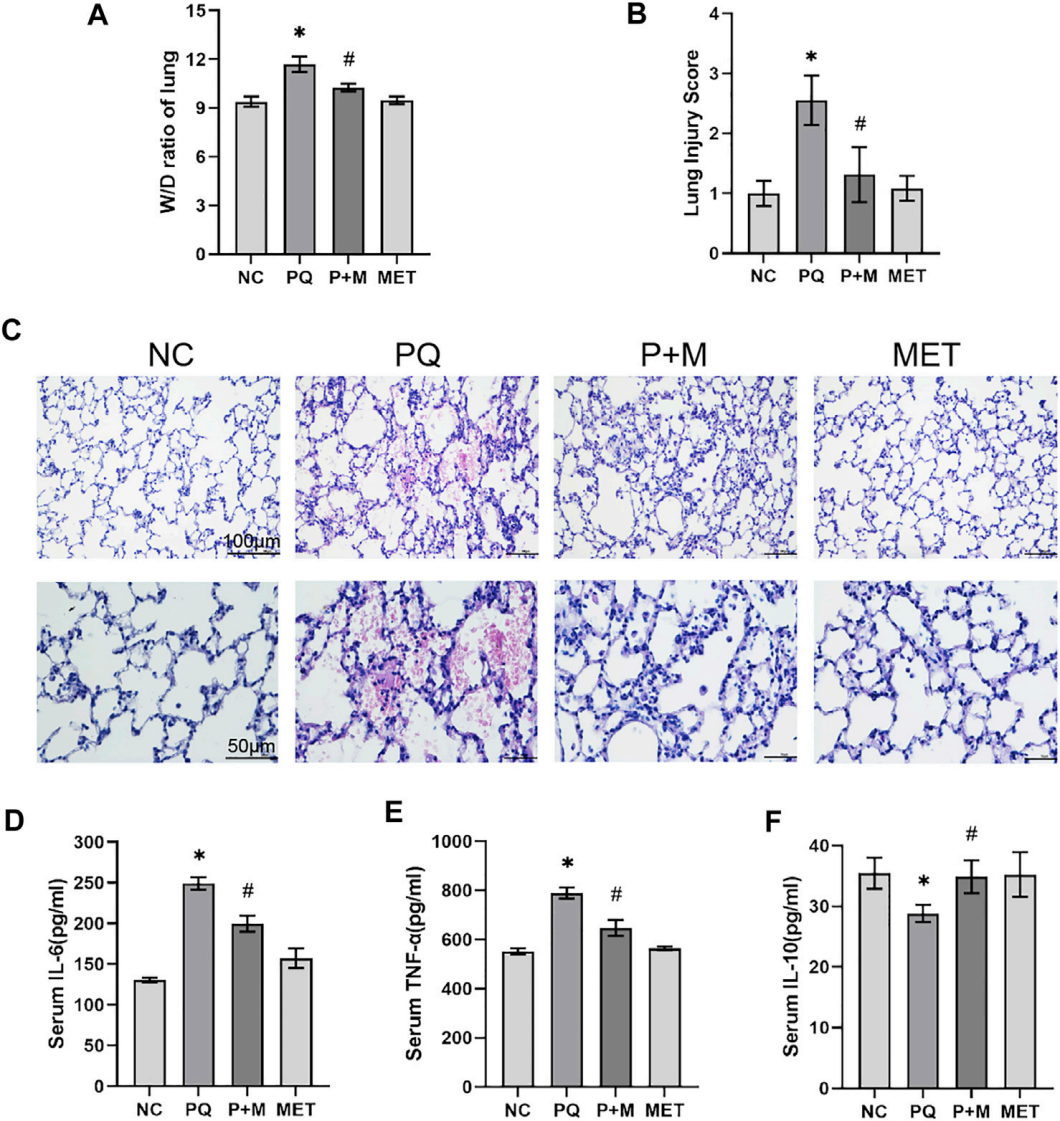
The effect of MET on ALI and systemic inflammatory response in PQ-poisoned rats. **(A)** Lung W/D ratio, **(B)** Lung injury score, **(C)** Lung HE staining, ELISA kit to detect the levels of IL-6 **(D)**, TNF-α **(E)**, and IL-10 **(F)** in the serum of rats in each group (*n* = 6). Compared with the NC group, ^*^ *p* < 0.05; compared with the PQ group, ^#^ *p* < 0.05.

Between the NC and PQ groups, the lung W/D ratio and lung injury score of rats were significantly higher (*p* < 0.05) in the PQ group than the NC group; compared with the PQ group, the lung W/D ratio and lung injury score of rats in the P + M group decreased significantly (*p* < 0.05) (Figures 2A,B). The lungs of rats in the NC group showed a normal lung structure without inflammatory cell exudation, bleeding, alveolar collapse, and alveolar wall thickening; the lungs of rats in the PQ group showed diffuse inflammatory cell exudation and extensive alveolar hemorrhage, a large number of alveolar collapse, and alveolar wall thickening; the lungs of rats in the P + M group showed inflammatory cell exudation, partial alveolar collapse, and alveolar wall thickening, without bleeding; and the lungs of rats in the MET group had normal lung structure (Figure 2C). As shown in Figures 2D–F, compared with the NC group, the levels of IL-6 and TNF-α in the serum of rats in the PQ group increased significantly, while the IL-10 levels decreased significantly (*p* < 0.05); compared with the PQ group, the IL-6 and TNF-α levels in the serum of rats in the P + M group were significantly decreased, and the IL-10 levels were significantly increased (*p* < 0.05).

### Metformin Regulated the Expression of Inducible Nitric Oxide Synthase and Arg1 in the Lungs of Paraquat-Poisoned Rats

**FIGURE 3:**
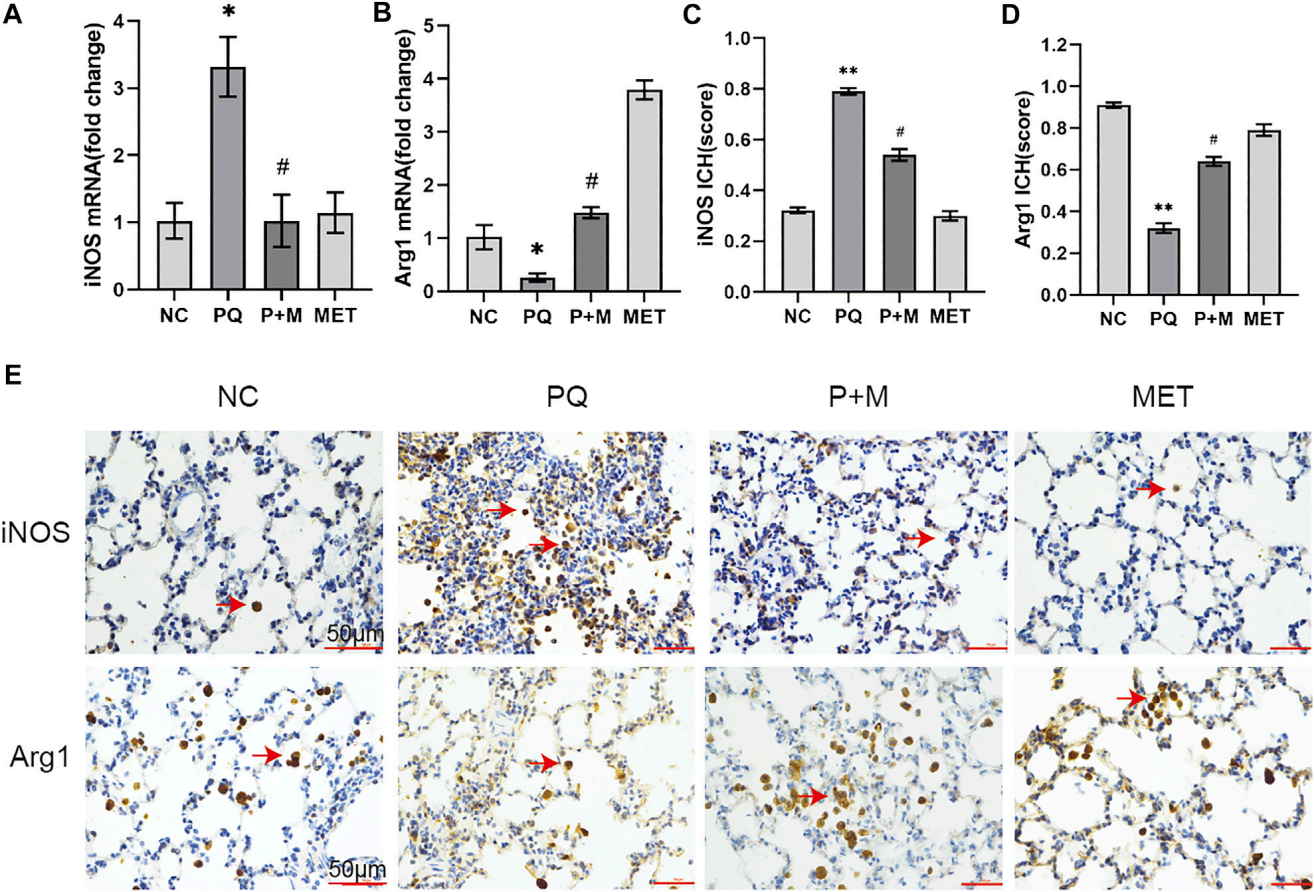
The effect of MET on the expression of iNOS and Arg1 in the lungs of PQ-poisoned rats. mRNA expression level of iNOS **(A)** and Arg1 **(B)** (*n* = 3), the semi quantitative analysis of immunohistochemical of iNOS **(C)** and Arg1 **(D)** (*n* = 3), **(E)** immunohistochemical of iNOS and Arg1 in lung tissue. Compared with the NC group, ^*^ *p* < 0.05, ^**^ *p* < 0.01; compared with the PQ group, ^#^ *p* < 0.05.

Figures 3A,Bshows the iNOS and Arg1 mRNA expressions in the NC and PQ groups. The iNOS mRNA expression in the lungs of the PQ group showed a significant increase than the NC group, while the Arg1 mRNA expression showed a significant decrease (*p* < 0.05) than the NC group. However, compared with the rats in the PQ group, the iNOS mRNA expression in the lung tissue of the rats in the P + M group reduced significantly, while the Arg1 mRNA expression increased significantly (*p* < 0.05). Immunohistochemistry was used to detect the expression of M1 marker of iNOS and M2 marker of Arg1 in lung tissue (E). The semi quantitative analysis of immunohistochemical results showed that the iNOS expression increased while the Arg1 expression decreased in the lungs of the PQ group compared to the NC group (*p* < 0.01); however, the iNOS expression decreased, and the Arg1 expressions increased in the lungs of the P + M group compared to the PQ group (*p* < 0.05) (Figures 3C,D).

### The Cytotoxic Effects of Metformin on NR8383 and Paraquat-Induced NR8383

**FIGURE 4:**
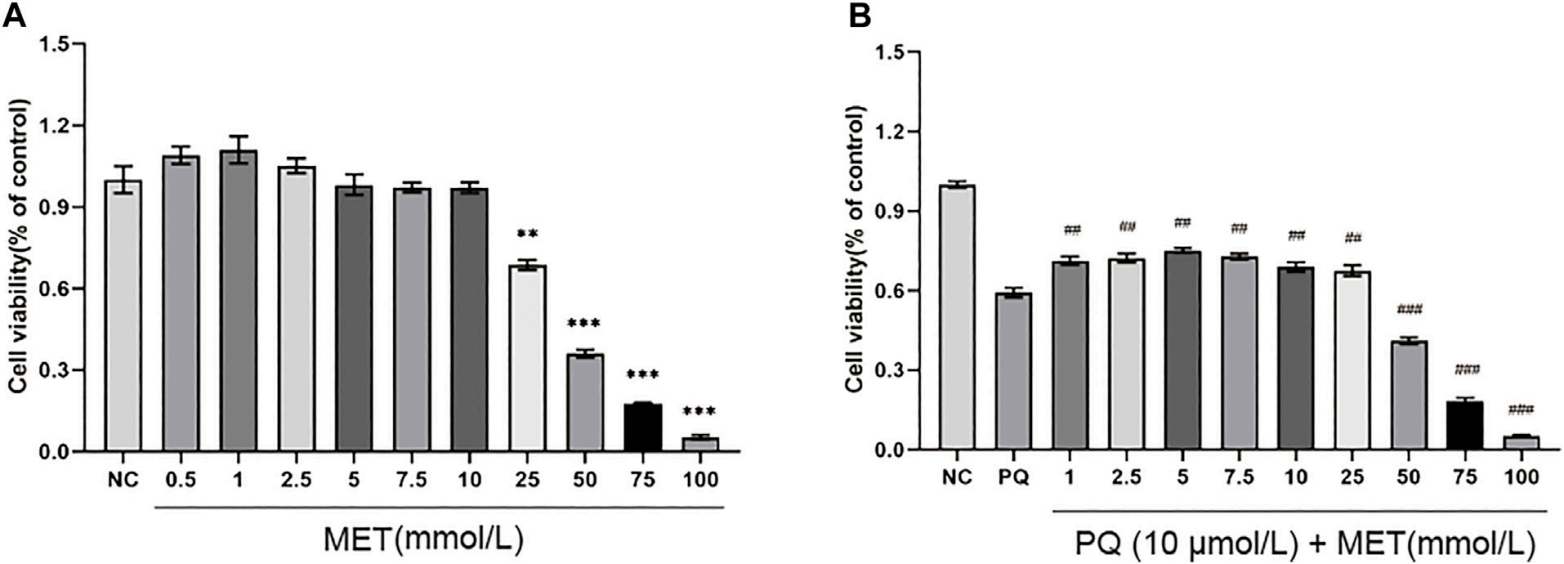
The effect of MET on the viability of NR8383. Different concentrations of MET (0.5, 1, 2.5, 5, 7.5, 10, 25, 50, 75, and 100 mmol/L) were used to interfere with **(A)** NR8383 and **(B)** PQ-induced NR8383 for 48 h, and the cell viability of each group was calculated. Compared with the NC group, ^**^ *p* < 0.01, ^***^ *p* < 0.001, compared with the PQ group, ^##^ *p* < 0.01, ^###^ *p* < 0.001.

Figure 4A shows different concentrations of MET interfered with NR8383 for 48 h. Compared with the NC group, MET (0.5–2.5 mmol/L) increased cell viability to a certain extent, but it was not statistically significant (*p* > 0.05); MET at a concentration of 5–10 mmol/L has no significant effect on cell viability (*p* > 0.05); however, MET concentrations of 25–100 mmol/L significantly reduced cell viability (*p* < 0.01). Figure 4B shows different concentrations of MET interfered with 10 μmol/L PQ-induced NR8383 for 48 h. Compared with the PQ group cells without MET intervention, MET at a concentration of 1–25 mmol/L increased cell viability to varying degrees, and there was a significant difference (*p* < 0.01); MET at a concentration of 50–100 mmol/L significantly reduced cell viability (*p* < 0.001).

### Metformin Regulated Paraquat-Induced Polarization of NR8383

**FIGURE 5:**
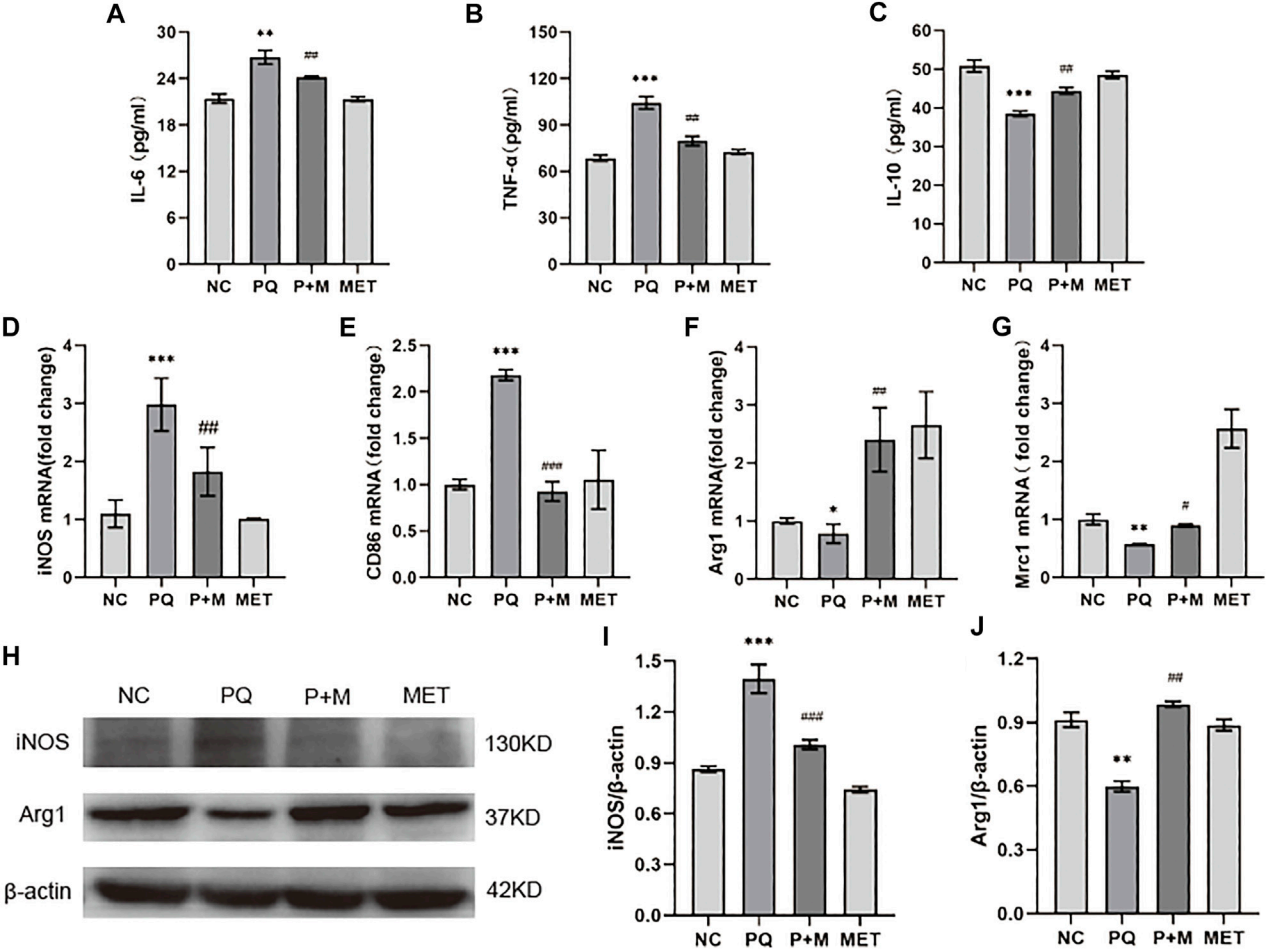
The effect of MET on the polarization of PQ-induced NR8383. ELISA kits detect the levels of IL-6 **(A)**, TNF-α **(B)**, and IL-10 **(C)** in the cell supernatant (*n* = 3); mRNA expression levels of iNOS **(D)**, CD86 **(E)**, Arg1 **(F)**, Mrc1 **(G)** in cells (*n* = 3); **(H)** protein expression levels of iNOS **(I)**, Arg1 **(J)** in cells (*n* = 3). Compared with the NC group, ^*^ *p* < 0.05, ^**^ *p* < 0.01, ^***^ *p* < 0.001; compared with the PQ group, ^#^ *p* < 0.05, ^##^ *p* < 0.01, ^###^ *p* < 0.001.

Compared with the NC group, the PQ group showed a significant increase in the pro-inflammatory factors, IL-6 and TNF-α, levels in the NR8383 supernatant, and a significant decrease in the anti-inflammatory factor IL-10 levels (*p* < 0.01); however, the addition of MET significantly decreased the IL-6 and TNF-α levels, and the IL-10 levels increased significantly (*p* < 0.01) (Figures 5A–C). The iNOS, CD86 mRNA, and iNOS protein levels in the PQ group were significantly higher than those in the NC group, while the levels of Arg1, Mrc1 mRNA, and Arg1 protein were significantly decreased (*p* < 0.05) (Figures 5D–G). Additionally, compared with the PQ group, the iNOS, CD86 mRNA, and iNOS protein levels of the P + M group were significantly decreased, while the Arg1, Mrc1 mRNA, and Arg1 protein levels increased significantly (*p* < 0.05) (Figures 5H–J).

## Discussion

The PQ production and sale has been banned in many countries; however, many people continue to die from PQ-induced ALI every year (Gao et al., 2021b; Chen et al., 2021). Exploring the effective treatment of PQ-induced ALI is still a clinical problem. The *in vivo* experiments showed that the 35 mg/kg MET increased the survival rate of the PQ-poisoned rats, and significantly alleviated the lung damage and systemic inflammatory response. Further studies have found that PQ can increase the alveolar macrophages’ polarization toward M1 and decrease toward M2, and MET could improve this PQ-induced effect.

We have found through animal experiments that MET can significantly increase the survival rate of rats. Clinically, the maximum recommended dose of oral MET for adult patients with type 2 diabetes is 2,550 mg per day (LaMoia and Shulman, 2021), when we administered MET intraperitoneally at a dose of 35 mg/kg, the survival rate of PQ-poisoned rats increased significantly. According to the animal and human drug conversion model (Nair et al., 2018), the intraperitoneal injection of 35 mg/kg of MET in rats is equivalent to the oral administration of 2000 mg per day for adults, which is a safe dose in clinical practices and provides a possibility for MET to be used in the treatment of patients with clinical PQ poisoning.

MET can reduce the systemic inflammatory response and the lung pathological damage in PQ-poisoned rats. Recent studies have found that MET plays a significant role in lowering blood sugar and anti-inflammatory and acute lung injury treatments (Chen et al., 2020a). Zhang et al., (2017) found that MET ameliorated LPS-induced lung injury and reduced the expression levels of IL-6 and TNF-α in rat bronchoalveolar lavage fluid. Wu et al., (2018) reported that MET reduced the IL-6 and TNF-α levels in the plasma and lung tissue homogenate of mice with LPS-induced ALI, alleviated the lung injury, and reduced the mortality of the mice. MET also alleviated lung pathology in animal models of ALI induced by particulate matter (PM) (2.5) and ventilator (Tsaknis et al., 2012; Gao et al., 2020). Our *in vivo* studies showed that MET reduced the IL-6 and TNF-α in the rat’s serum, increased the expression level of IL-10, and significantly alleviated the lung pathological damage in PQ-poisoned rats, similar to the results of Wu et al., (2019).

Our study found that MET ameliorated PQ-induced ALI in rats, decreased the expression of M1 macrophage marker iNOS in the lungs, and increased the M2 macrophage marker Arg1, which suggested us that MET may attenuate the polarization of lung macrophages toward M1, and strengthened the M2 polarization. Static macrophages can be activated by LPS, interferon γ, and so forth, into M1 type by secreting pro-inflammatory factors, such as IL-1β, TNF-α, and iNOS, to promote continuous inflammation and tissue damage; it can also be activated by IL-4 or IL-13 into M2 type, reducing inflammation and repairing the tissues by secreting anti-inflammatory factors, such as IL-10, TGF-β, and Arg1 (Shapouri-Moghaddam et al., 2018). Kim et al., (2014) found that MET inhibited the secretion of TNF-α and IL-6 in an LPS-induced RAW264.7 in a concentration-dependent manner. Kelly et al., (2015) found that MET promoted the IL-10 secretion in primary macrophages of mouse bone marrow induced by LPS in a dose-dependent manner. Our research also found that MET inhibited the IL-6 and TNF-α secretion in the PQ-induced NR8383, and promoted the IL-10 secretion. Our results were consistent with the results of previous literature, although the cell lines and intervention drugs were different. The release of macrophage inflammatory factors and their polarization direction are inseparable (Shapouri-Moghaddam et al., 2018). Jing et al., (2018) found that MET reduced the IL-6 and TNF-α secretion in palmitate-stimulated RAW264.7 and mouse bone marrow primary macrophages; the MET treatment reduced the M1 macrophages proportion and increased the M2 macrophages proportion. Our results also confirmed that MET intervention attenuated PQ-induced NR8383 polarization to M1 and promoted its polarization to M2.

Additionally, our research results suggested that MET at a concentration greater than 10 mmol/L was toxic to NR8383, and the toxicity is dose-dependent. Mitochondria are the main subcellular target of MET, inhibiting the mitochondrial complex I (Fontaine, 2018). However, excessive MET destroys the mitochondrial function and hinders the normal energy metabolism of cells (Chowdhury et al., 2020), suggesting a relationship between cell death induction and high MET concentrations. The specific cause of death of NR8383 caused by high concentration of MET and the specific mechanism by which MET regulates the polarization of NR8383 are our next research direction.

## Conclusion

Our study found that MET increased polarization of alveolar macrophages to the anti-inflammatory M2 type, decreased polarization to the pro-inflammatory M1 type, alleviated the lung injury and systemic inflammatory response in rats with PQ poisoning, thereby reducing the mortality of PQ-poisoned rats and opened up new ideas for the PQ-induced ALI treatment and has important clinical significance.

## Data Availability

The raw data supporting the conclusions of this article will be made available by the authors, without undue reservation.
