# Supplementary material for: Metformin Regulates Alveolar Macrophage Polarization to Protect Against Acute Lung Injury in Rats Caused by Paraquat Poisoning
Source: Front Pharmacol. 2022 May 13;13:811372. doi: 10.3389/fphar.2022.811372 (PMC9136134; doi:10.3389/fphar.2022.811372)
Supplement: Supplementary file 1 [file Table1.DOCX]

Table 1 Primer sequences used for qRT-PCR.

| mRNA | Primers | Sequences (5′–3′) |
| --- | --- | --- |
| iNOS | Upstream | AGACGCACAGGCAGAGGT |
|  | Downstream | AGGCACACGCAATGATGG |
| Arg1 | Upstream | GTGAAGAACCCACGGTCTGT |
|  | Downstream | GCCAGAGATGCTTCCAACTG |
| CD86 | Upstream | GCTCTCAGTGATCGCCAAC |
|  | Downstream | TCTTTGTAGGTTTCGGGTATC |
| Mrc1 | Upstream | CTTCGGGCCTTTGGAATAAT |
|  | Downstream | TAGAAGAGCCCTTGGGTTGA |
| β-actin | Upstream | ACTATCGGCAATGAGCGGTTCC |
|  | Downstream | CTGTGTTGGCATAGAGGTCTTTACG |
