# Supplementary material for: Metformin Regulates Alveolar Macrophage Polarization to Protect Against Acute Lung Injury in Rats Caused by Paraquat Poisoning
Source: Front Pharmacol. 2022 May 13;13:811372. doi: 10.3389/fphar.2022.811372 (PMC9136134; doi:10.3389/fphar.2022.811372)

experimental design

In order to better understand the experimental process, we made an experimental design.

1.This figure describes how we conducted survival experiments in rats.

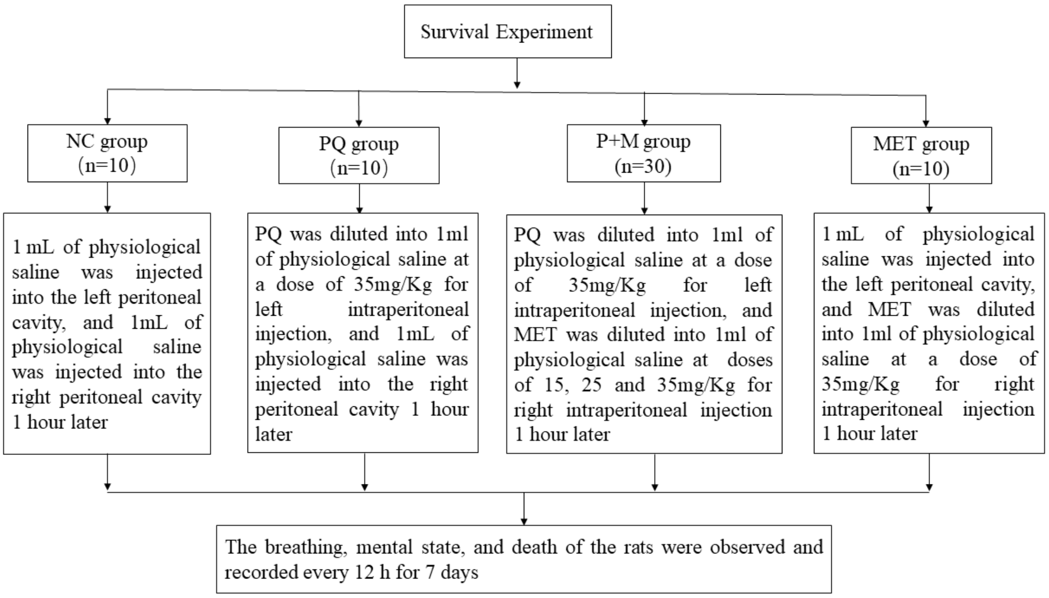

2.This figure describes how we performed the experimental process of metformin in the treatment of acute lung injury in rats caused by paraquat poisoning.

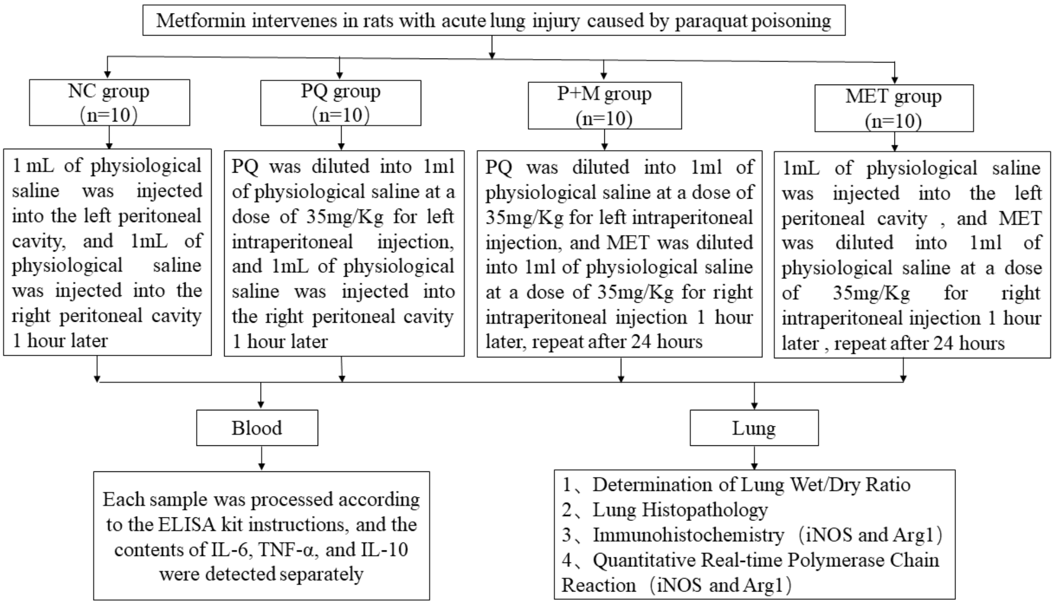

3.This figure describes how we selected the intervention concentration of metformin in *vitro* experiments.

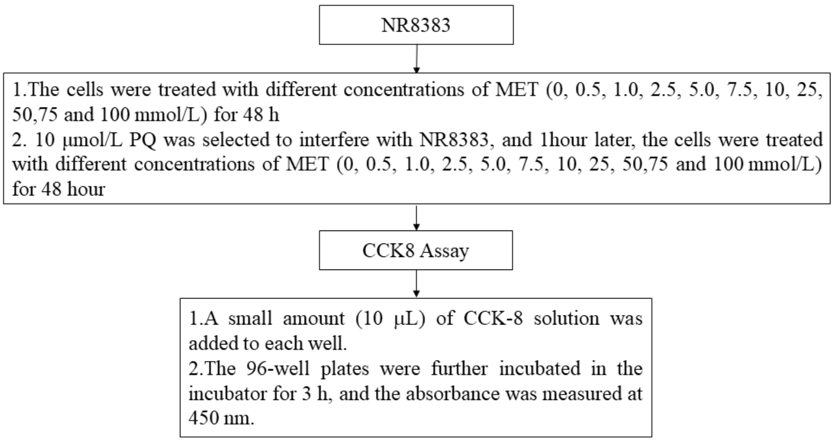

4.This figure describes how we investigated the effect of metformin on paraquat-induced polarization of NR8383 in *vitro* experiments.

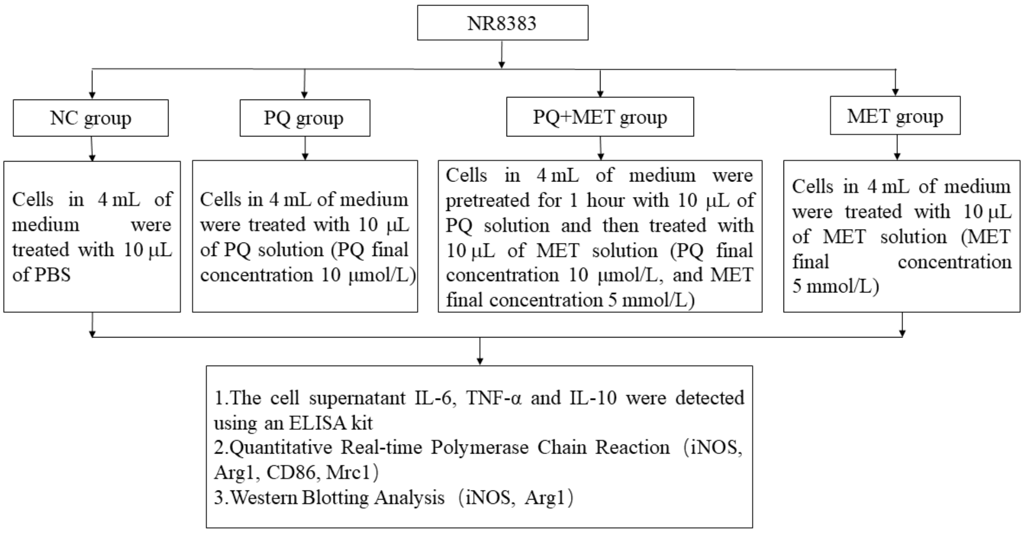

Supplement: Supplementary file 2 [file DataSheet1.PDF]
